# Utilizing distributed acoustic sensing and ocean bottom fiber optic cables for submarine structural characterization

**DOI:** 10.1038/s41598-021-84845-y

**Published:** 2021-03-10

**Authors:** Feng Cheng, Benxin Chi, Nathaniel J. Lindsey, T. Craig Dawe, Jonathan B. Ajo-Franklin

**Affiliations:** 1 Department of Earth, Environmental, and Planetary Sciences, Rice University, 6100 Main Street, Houston, TX 77005 USA; 2 Energy Geosciences Division, Lawrence Berkeley National Laboratory, 1 Cyclotron Road, Berkeley, CA 94720 USA; 3 Geophysics Department, Stanford University, 397 Panama Mall, Stanford, CA 94305 USA; 4 Monterey Bay Aquarium Research Institute, 7700 Sandholdt Road, Moss Landing, CA 95039 USA

**Keywords:** Seismology, Physical oceanography, Geophysics

## Abstract

The sparsity of permanent seismic instrumentation in marine environments often limits the availability of subsea information on geohazards, including active fault systems, in both time and space. One sensing resource that provides observational access to the seafloor environment are existing networks of ocean bottom fiber optic cables; these cables, coupled to modern distributed acoustic sensing (DAS) systems, can provide dense arrays of broadband seismic observations capable of recording both seismic events and the ambient noise wavefield. Here, we report a marine DAS application which demonstrates the strength and limitation of this new technique on submarine structural characterization. Based on ambient noise DAS records on a 20 km section of a fiber optic cable offshore of Moss Landing, CA, in Monterey Bay, we extract Scholte waves from DAS ambient noise records using interferometry techniques and invert the resulting multimodal dispersion curves to recover a high resolution 2D shear-wave velocity image of the near seafloor sediments. We show for the first time that the migration of coherently scattered Scholte waves observed on DAS records can provide an approach for resolving sharp lateral contrasts in subsurface properties, particularly shallow faults and depositional features near the seafloor. Our results provide improved constraints on shallow submarine features in Monterey Bay, including fault zones and paleo-channel deposits, thus highlighting one of many possible geophysical uses of the marine cable network.

## Introduction

The detailed structure of seismogenic marine faults remain enigmatic in many regions, particularly those with minimal coverage by modern 3D reflection seismic surveys. This is doubly true with respect to temporal perturbations and related natural seismicity for events below the minimum detection threshold for on-shore seismic networks. These features, as well as seafloor mass transport processes such as landslides and turbidity currents, present significant geohazards for marine infrastructure including pipelines and marine telecommuications cables^1,2^. While significant research has contributed to identifying the seismic properties, architecture, and hazard of fault zones in terrestrial settings^3–5^, marine faults are often embedded in complicated environments with subsurface structural features of other origins^6^ and are more challenging to evaluate.

The dynamic aspects of these marine hazards are the most problematic to characterize, even with the utilization of modern geophysical techniques^7^, due to the high cost of effectively “instrumenting the ocean”. Passive seismic acquisition in marine environments is logistically difficult; the primary acquisition approach is the use of nodal ocean bottom seismometer (OBS) arrays with limited operating periods, no telemetry, and the requirement of return trips for retrieval. An alternative instrumentation strategy for targeted domains are cabled 4C short-period seismometer arrays, sometimes used for life-of-field monitoring in oil and natural gas production^8^. While this approach has provided a rich array of results, particularly for 4D mapping of fluid movement^9–11^, the high deployment costs are prohibitive for most scientific studies.

Fault zones have a range of geophysical properties which can be exploited for identification. Lower seismic velocities in fault zones, particularly those which have experienced substantial historical slip, have been identified through lateral guided mode measurements^4,12,13^, resonance studies^14^, and imaging approaches such as refraction tomography^15,16^. Recent active source studies have also attempted to utilize scattered surface waves to identify near-surface fault complexes^17,18^. In these cases, coherent scattered Rayleigh waves can be mapped back to scattering locations to provide high resolution constraints on lateral property contrasts.

Ambient noise processing techniques^19,20^ can provide a powerful tool for performing structural imaging of faults^16,21,22^ while simultaneously recording small seismic events with high density passive seismic arrays^23,24^. Given the challenges of performing “large N” marine passive seismic acquisition, submarine fiber optic cables, which cross an increasing number of offshore locations, present the possibility for marine passive seismic measurements based on the recently-developed distributed acoustic sensing (DAS) technique. DAS utilizes an interrogator unit (IU) to launch short laser pulses along a fiber optic cable and samples high spatial and temporal resolution dynamic strain perturbations by measuring phase changes in the Rayleigh backscattered light^25^ and has found broad application in both passive and active source seismology^26–32^. At present, there are over 350 active submarine cables spanning 1.2 million kilometers connecting very close to 100 countries (TeleGeography^33^). As shown by two recent studies^34,35^, DAS offers the capacity to turn these global cables into a powerful sensing resource if appropriate analysis tools are utilized, providing a path towards characterizing previously hidden offshore structures.

**Figure 1:**
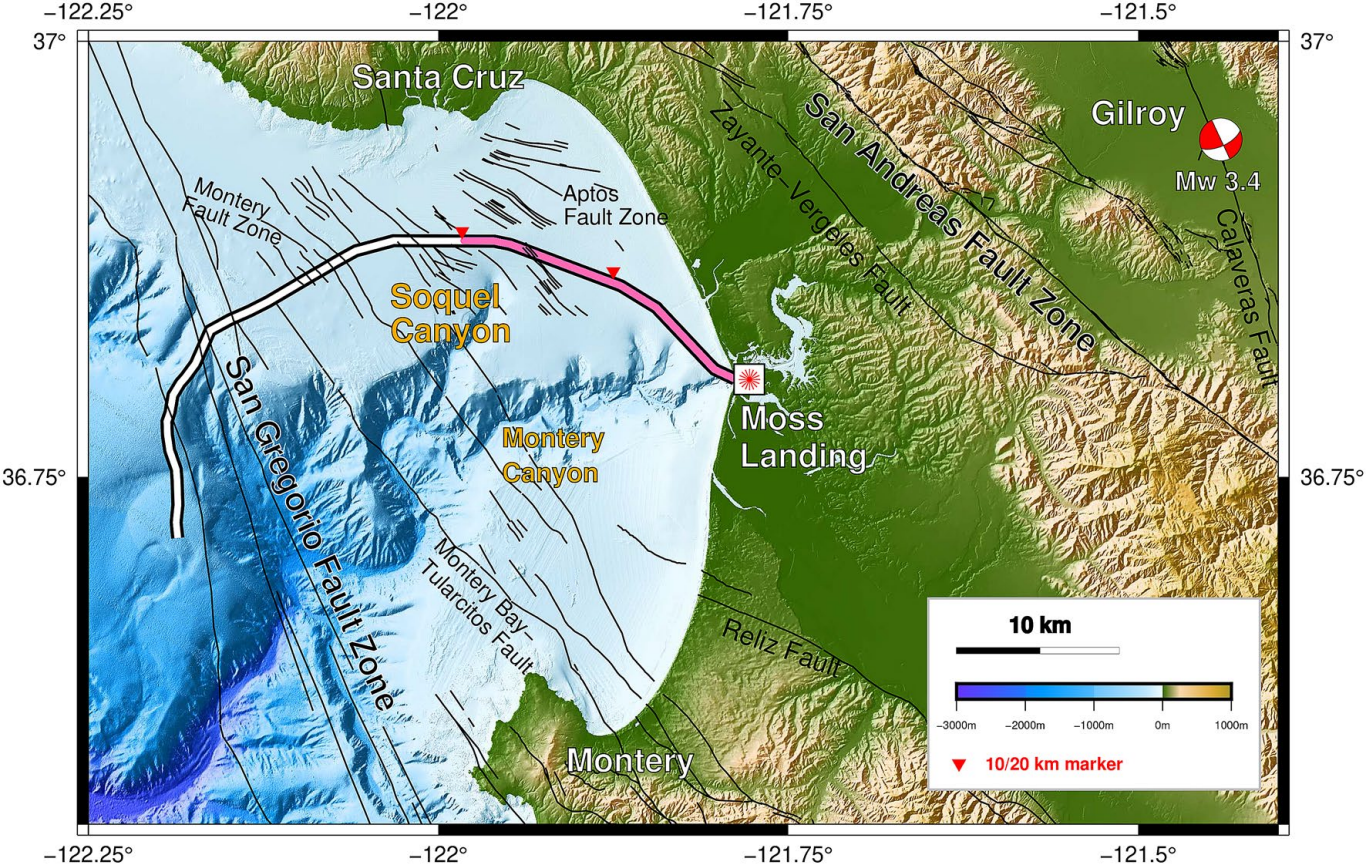
MARS DAS experiment. Map of Monterey Bay, CA, showing the MARS cable (DAS, pink portion), mapped faults, the Gilroy earthquake (red-and-white beach ball), and major bathymetric features.

Our study utilizes a marine DAS dataset from Monterey Bay first discussed in^34^, acquired north of Monterey Canyon; this near-shore environment highlights a rich array of processes including active tectonics associated with the San Andreas fault system as well as rapid channel erosion and deposition. Contemporary and historical channel and mass transport systems are fed by sediments from the Salinas and Pajaro Rivers^36^. Recent high-resolution 2D reflection seismic studies^37^ have also identified and mapped paleo-channel deposits associated with earlier geometries of both the Monterey and Soquel canyons. In turn, the orientation of these systems may be partially controlled by deeper fault lineaments, yet to be effectively constrained with available data. These channel systems incise the Miocene to Pleistocene Purisma formation^38,39^ which is diffusely faulted. While the DAS profile we investigate does not cross the San Gregorio fault which is farther offshore or the San Andreas (onshore), it does crossed mapped sections of the Aptos Fault Zones (AFZ) and approaches the eastern edge of Monterey Bay Fault Zones (MBFZ). An imaging challenge in this context is the superposition of recent, and presumably low velocity, channel fill materials in the overburden with deeper altered fault structures. Fig. 1 provides the geological context for the study.

**Figure 2:**
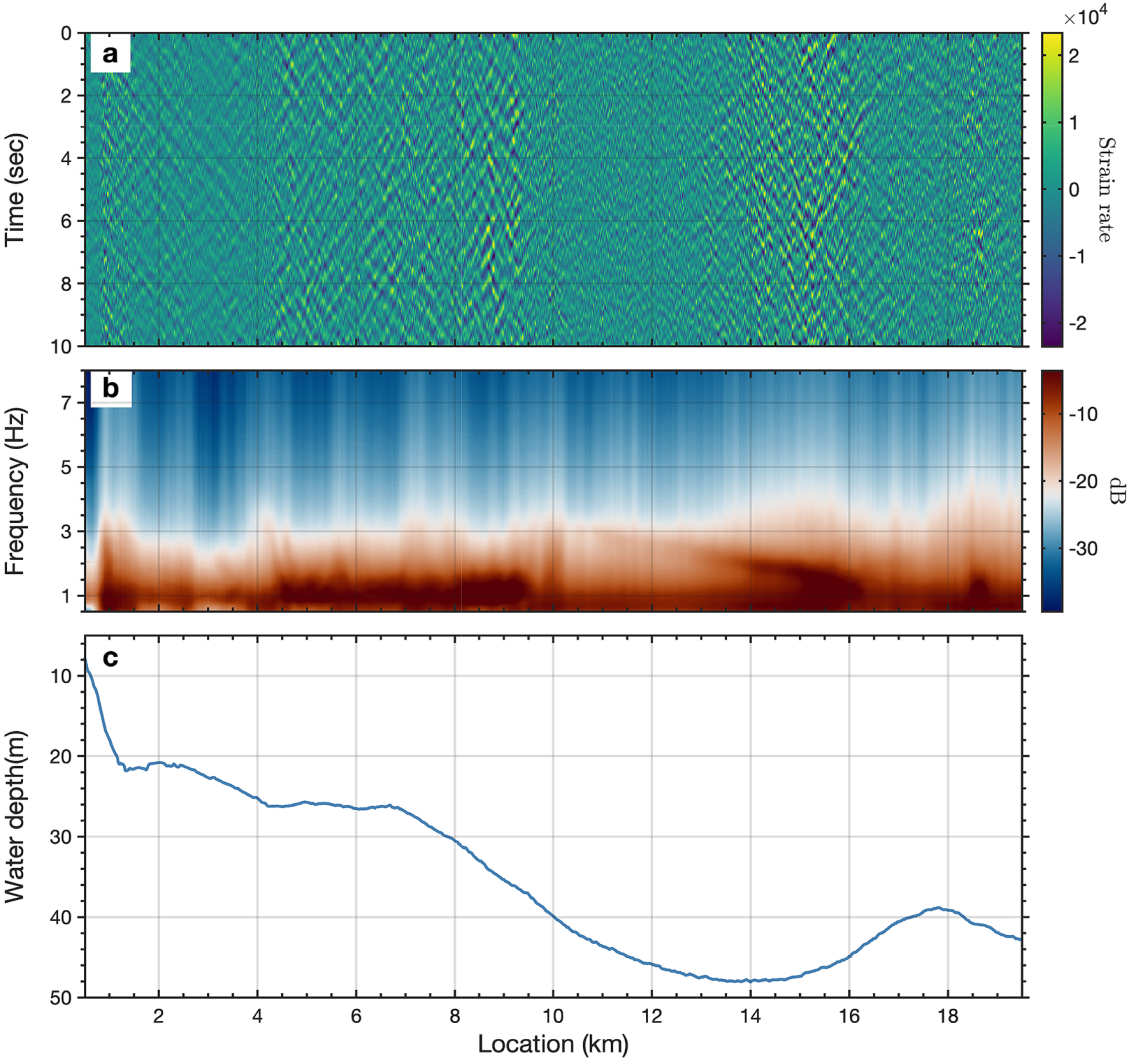
Observations of oceanic microseism noise. (**a**) 10-second-long oceanic microseism noise record of strain-rate (unit, *nanostrain*/*s*) along the 20 km fiber optic cable. (**b**) 4-day averaged spectrum of the noise along the cable. We convert strain-rate into strain for the spectral density measurement. (**c**) Sea water depth along the cable.

In this study, we investigate a sequence of seismic features which we believe are small faults zones and previously mapped paleo-channel units. We analyse continuous DAS strain-rate data along a 20 km section of a 51 km long optical cable over 4 days in March 2018. Prior analysis of this dataset revealed multiple zones where seismic conversions occurred, some of which were co-located with existing faults, and thus these zones were presumed to be caused by wavefield interaction with seafloor faults. Here we utilize ambient noise interferometry techniques to further probe the characteristics of these zones. Our aim is to improve understanding of the internal shear wave velocities ($V_s$) and scattering properties of these zones use these measurements to place them in a regional geologic context. We first retrieve empirical Green’s functions (EGFs) which show characteristic coherent Scholte waves, P-SV polarised waves near the fluid–solid interface, over several kilometers with appropriate dispersion properties. We invert these data from $0.75\sim 5 Hz$ and generate a depth-resolved image of near-seafloor structure encompassing the top 400 m of the seabed. The EGFs also show evidence of coherently scattered Scholte waves. We migrate the scattered wavefield using two different techniques to better localize the scattering features. These observations, coupled with shear wave inversions and interpretive forward modeling of scattering response, provide improved constraints on these zones, which are likely a combination of faulting and paleo-channel deposits, and highlight one of many possible geophysical uses of the marine cable network.

**Figure 3:**
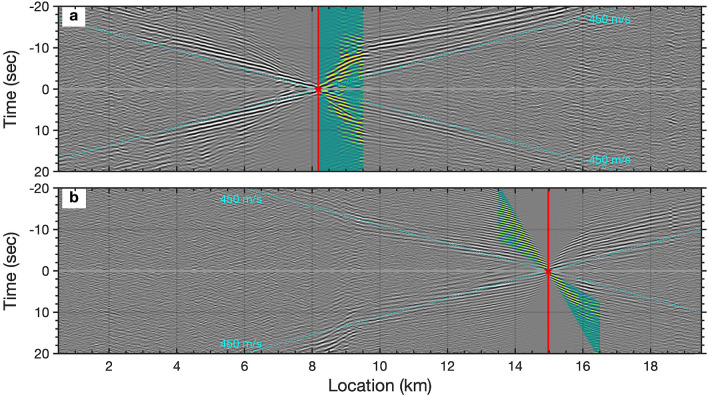
Scholte waves retrieved from oceanic microseism noise along the 20 km cable. (**a**,**b**) show empirical Green’s function gathers with virtual sources located at 8.2 km and 15 km, respectively. The red stars indicate the virtual sources. The cyan dashed lines indicate the approximate velocity of the Scholte wave. Backscattered Scholte waves are visible near the 9 km location of (**a**). The coherent signals on (**b**) appear to have a higher frequency which is consistent with the increasing spectrum on 2b.

**Figure 4:**
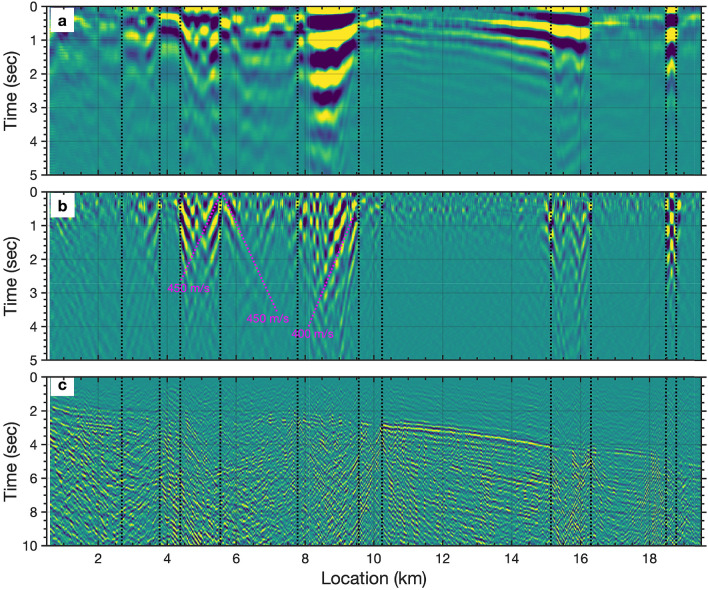
(**a**) Autocorrelation image from oceanic microseism noise. (**b**) The separated scattered Scholte waves from autocorrelation profile. (**c**) Magnitude 3.4 Gilroy earthquake wavefield observed on DAS cable on 11 March 2018. (A) The black dashed lines indicates the observed horizontal discontinuities. The fuchsia dashed lines indicate the apparent velocities of the scattered arrivals.

## Results

### Experiment overview and context

The existing Monterey Accelerated Research System (MARS) science cable spanning the continental shelf offshore of California (Fig. 1) was occupied for a four-day period of DAS observation beginning March 10th of 2018. A Silixa iDAS v2 interrogator unit was connected to one end of the fiber at the shore terminus of the MARS cable. The DAS method^40^, utilizes coherent pulses of laser light emitted through one single-mode fiber inside the cable, and measures optical phase changes in the backscattered signal. These phase changes are generated by local extension and contraction of the fiber induced by seismic waves or other sources; they were continuously recorded providing a passive record of the associated strain or strain-rate in the longitudinal direction. The recording consisted of a $\sim$10,000-channel, 20-km-long, single-component, strain-rate DAS dataset. These data were first reported in^34^, demonstrating the potential for using marine DAS for regional seismic event detection and potentially fault zone measurements. We further extend these observations by utilizing ambient noise DAS data to more definitively characterize seafloor structure.

**Figure 5:**
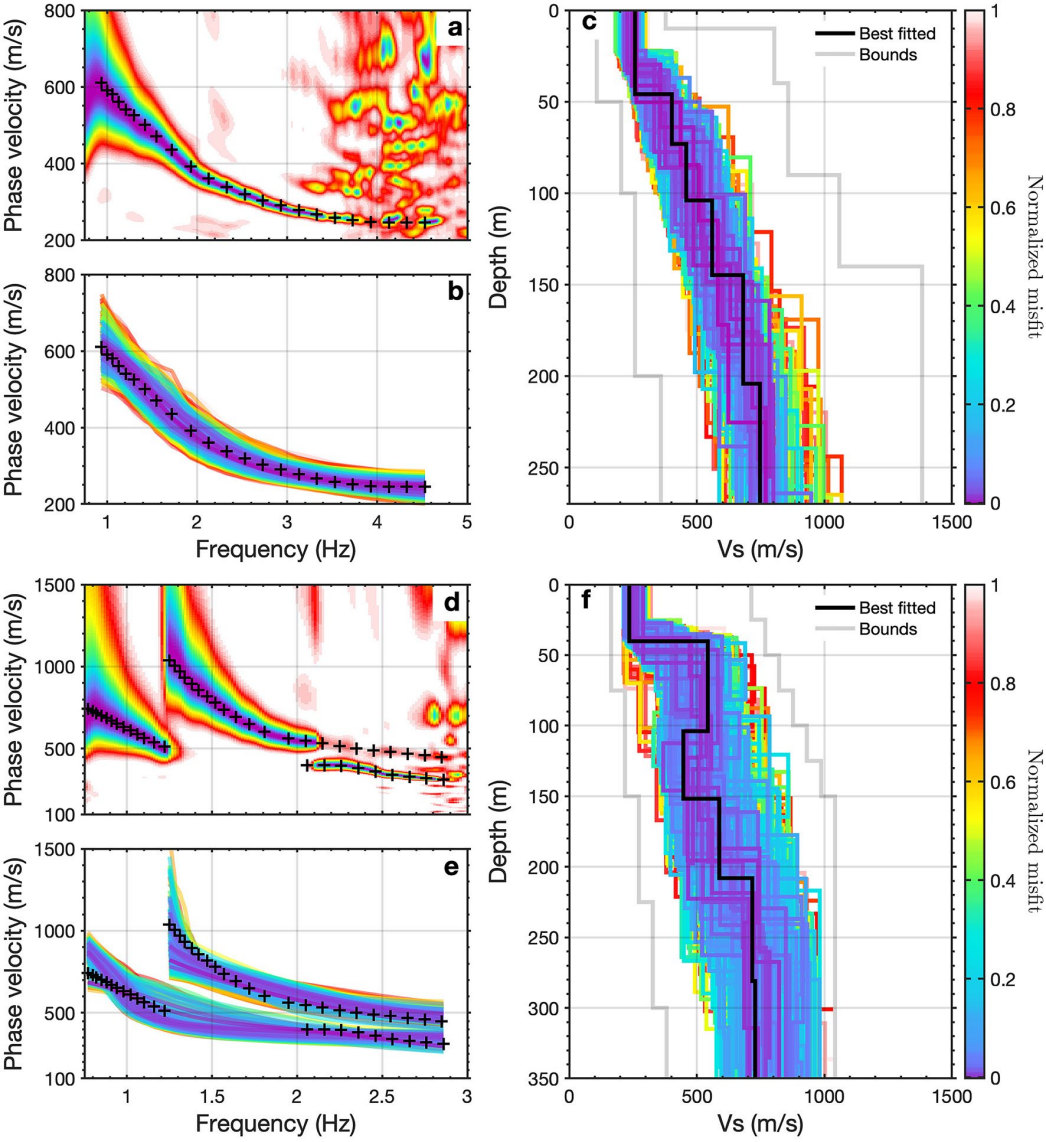
Examples of Scholte wave dispersion measurements and inversion. (**a**) Measured dispersion measurement and the picked dispersion curve with virtual source located at 6 km location; (**b**) presents the accepted forward modeled dispersion curves that fit measured dispersion curves well; (**c**) presents the accepted inverted $V_s$ models with the best fit model indicated by the solid line. (**d**–**f**) present the similar dispersion measurement with overtones and inversion with virtual source located at 17 km location.

**Figure 6:**
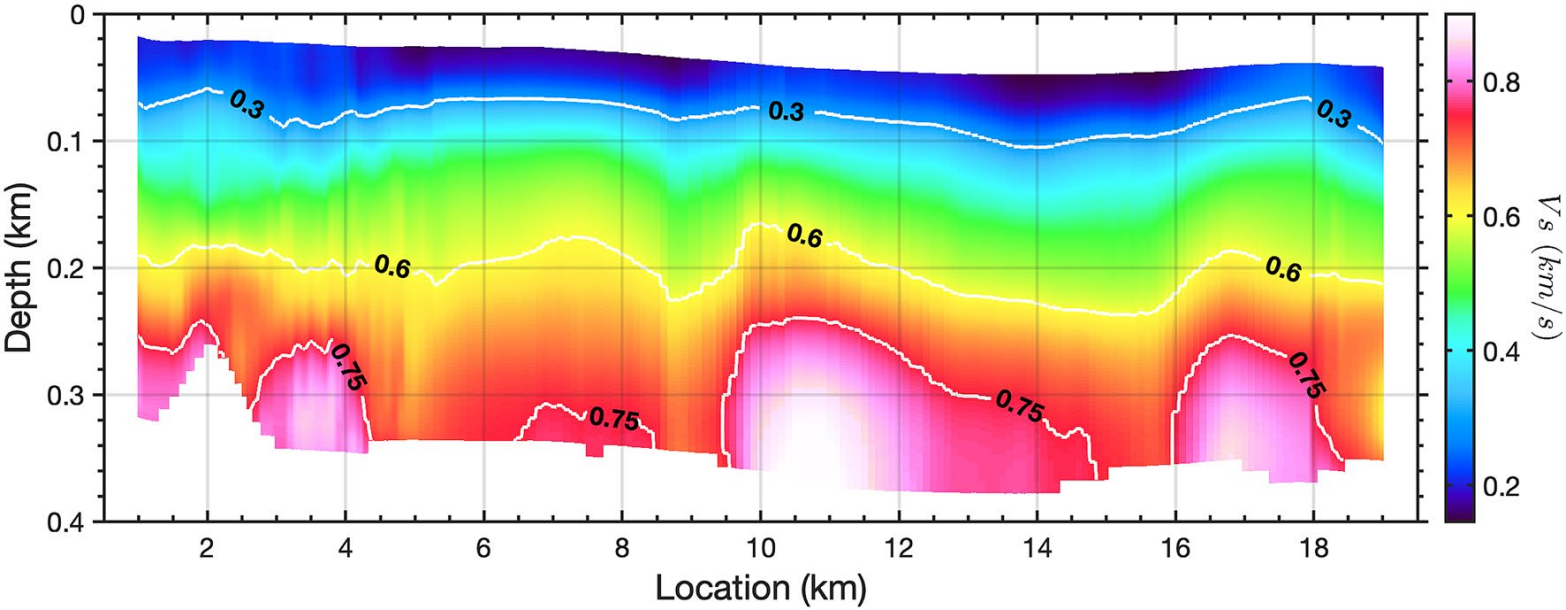
2D $V_s$ profile constructed from 1D $V_s$ models obtained from 181 sub-arrays of 1 km length. A $1\%$-width smoothing factor has been applied on the $V_s$ image along the profile. Shear-wave velocity model contours are shown in units of km/s.

### Coherent Scholte wavefields

Observations of ocean surface gravity waves and Scholte (P-SV solid-liquid interface) waves from marine DAS records have been recently reported by^34,35,41,42^. However, the raw strain-rate records of DAS (Fig. 2) are complicated by the superposition of a variety of coherent signals dominated by different frequency components, as well as incoherent and optical noise effects, e.g., temperature drift, interrogator unit shake, coupling issues. We apply ambient noise interferometry techniques^20,43^ to extract the coherent signals from the ambient DAS records (see Methods). Fig. 3 shows the retrieved empirical Green’s functions, sampled along a 20 km section of the fiber optic cable, for virtual sources located at 8.2 km (Fig. 3a) and 15 km (Fig. 3b), respectively. Clearly visible Scholte waves, surface waves propagating along the seafloor interface, can be seen with apparent velocities near 450 m/s. The time-distance view of the retrieved coherent signals wavefield, rather than the noise wavefield itself, provides a more intuitive view of the kinematics of seismic waves propagating along the cable. An animated image for all available virtual source gathers has been included in the Supplementary section.

**Figure 7:**
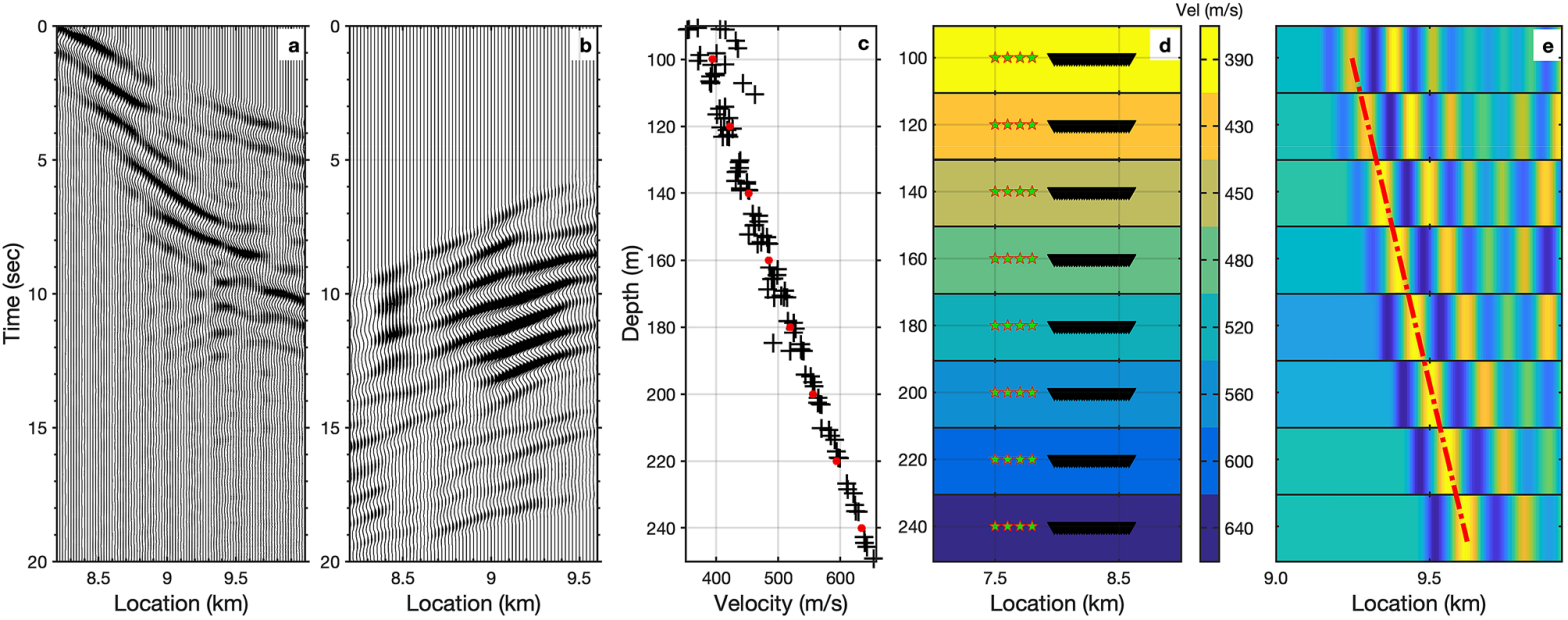
Kirchoff mapping of backscattered surface waves around location 9.5 km. (**a**) Retrieved forward-propagating Scholte wave from ambient noise interferometry with virtual source located at 8.2 km; (**b**). the separated backscattered Scholte waves after FK filtering; (**c**). the converted depth(wavelength)-velocity relationship from the measured dispersion curves using $depth = 0.4*v/f$; The dispersion curves used for depth(wavelength) conversion are picked from nearby 9 virtual source gathers. (**d**). earth models and source-receiver configuration for Kirchoff migration; the earth models are re-sampled from the converted depth(wavelength)-velocity relationship as indicated by the red dots. (**e**). the Kirchoff mapping image for scatters/heterogeneities localization at each depth. The red dash-dotted line represents the interpreted fault location.

**Figure 8:**
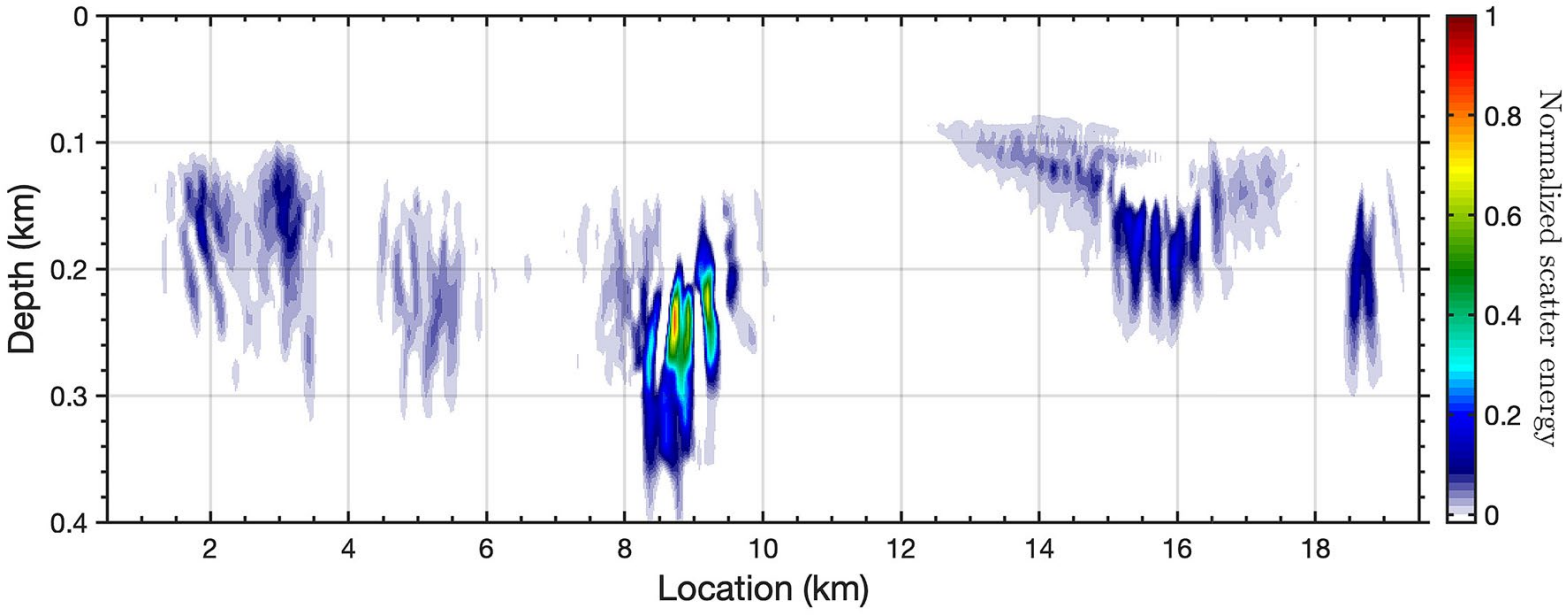
Image of Scholte wave scattering based on the natural migration technique.

Local discontinuities, due to lateral heterogeneity beneath the seabed, e.g., submarine faults, are also visible. A portion of the propagating wavefield is backscattered around 9 km (highlighted on Fig. 3a) indicating a potential laterally abrupt feature at this position^44^. Higher mode Scholte waves emerge in the off-shore section with higher frequency components and higher apparent velocities (highlighted on Fig. 3b) compared with the fundamental mode in the near-shore section (Fig. 3a).

### Scattering analysis from ambient noise DAS data

Ambient noise autocorrelation techniques have been successfully applied to image subsurface structure on both the Earth and Mars^45,46^, and have recently been used with DAS data offshore the Sanriku coast of Japan to image marine sediment thickness and velocity properties^35^. We obtain autocorrelation (zero offset cross-correlation) functions along the densely sampled DAS array (Fig. 4a), as by-products of ambient noise cross-correlation. Source wavelet effects have been minimized by median filter (using a $10\%$ running window). The resulting autocorrelation profile (Fig. 4a) indicates a distinct lateral variation along the 20 km cable with high spatial resolution (20 m). At this point, we are not confident that the autocorrelation horizons should be interpreted as specular reflections as suggested in past studies^35^. However, we can identify several boundaries as indicated by the dashed line on the the profile. These transitions in character likely coincide with lateral discontinuities in submarine structure. We note that several low velocity ($< 500 m/s$) scattered events exist around the discontinuity boundaries (Fig. 4a).

**Figure 9:**
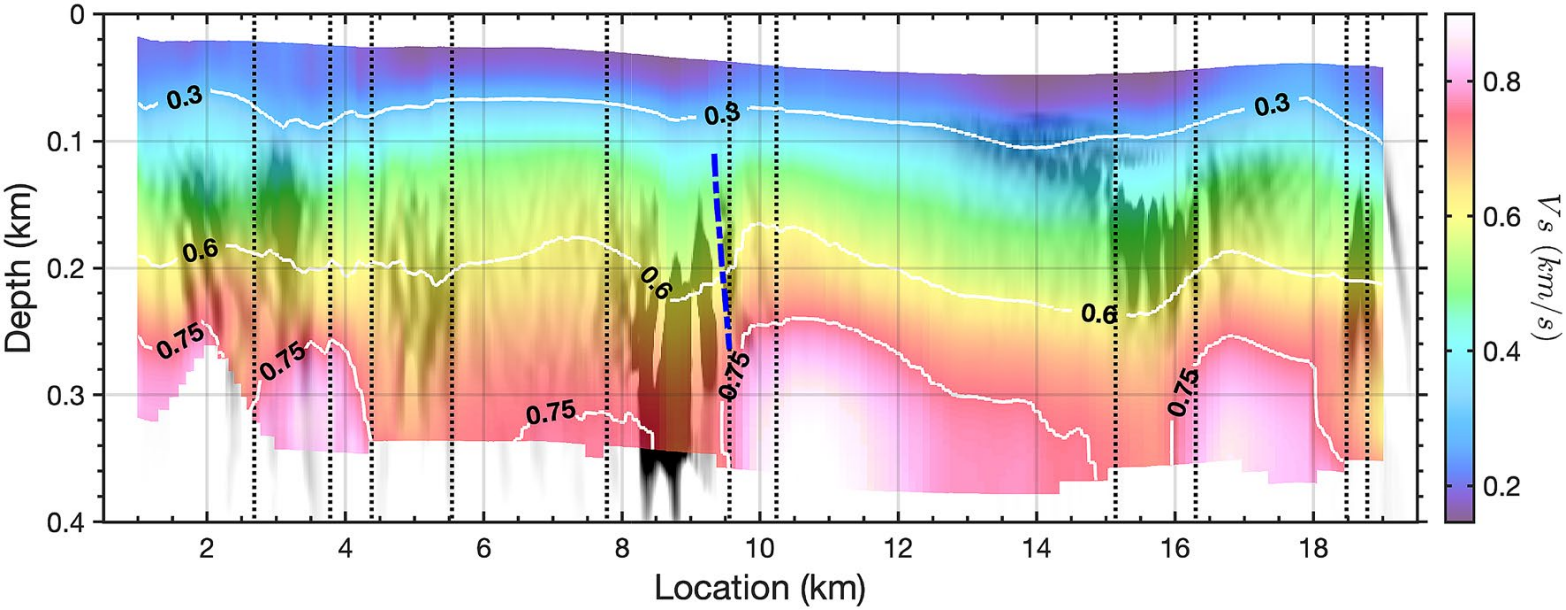
Integrated results using $V_s$ inversion and backscattered Scholte wave migration. The background gray image shows the natural migration result; the front color image shows the $V_s$ inversion profile; the blue dashed line represents the Kirchoff migration result. The black dashed lines indicate the observed horizontal discontinuity from autocorrelation image. Shear-wave velocity model contours are shown in units of km/s.

**Figure 10:**
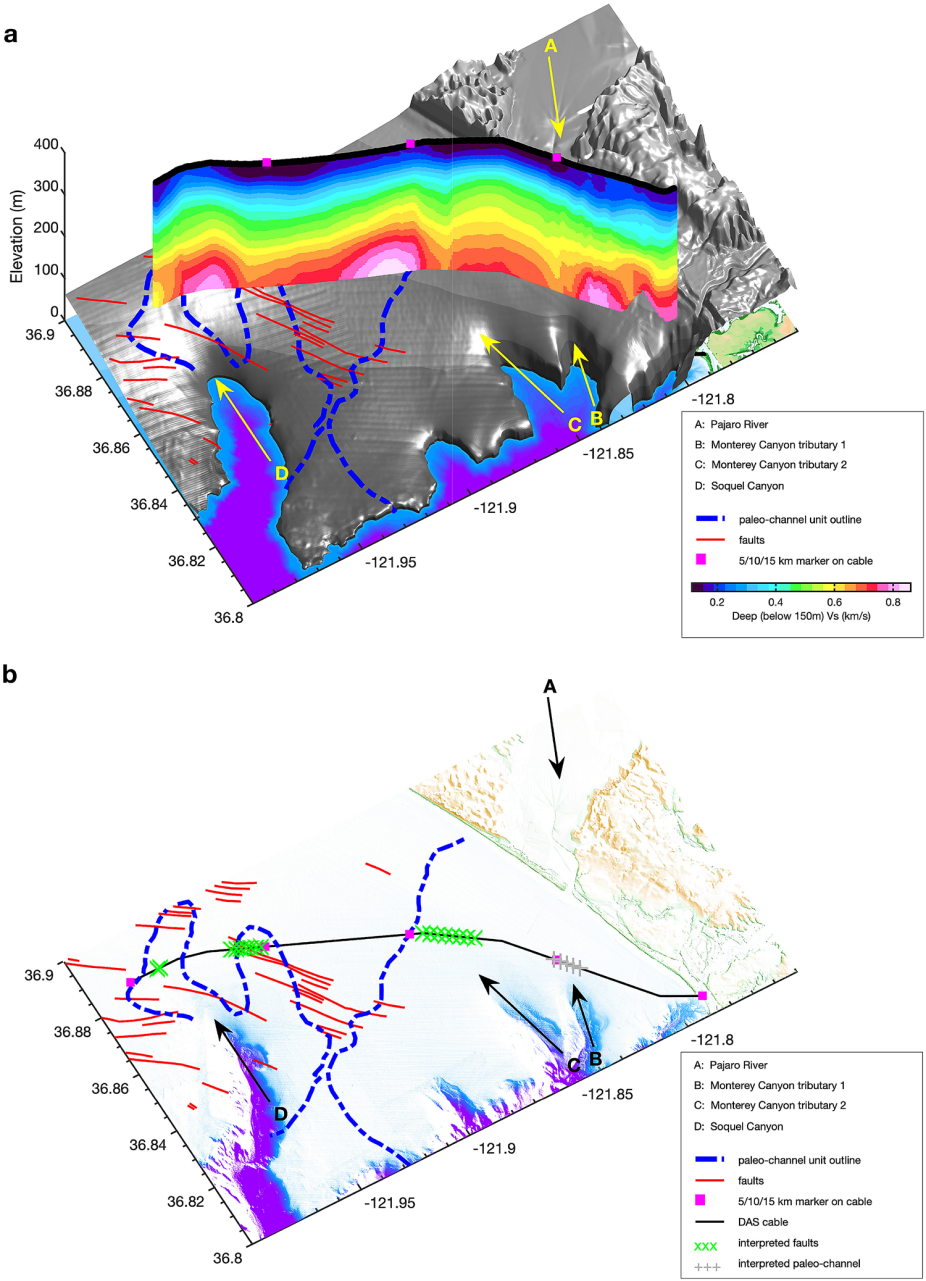
Integrated interpretation including submarine structural features. The vertical color image shows the inverted $V_s$ profile (Fig. 6); three pink squares on the $V_s$ profile indicate marks for cable locations (5 km, 10 km, 15 km); the red lines represent the mapped faults after^68^; the blue dashed lines indicate the outline of paleo-channel units obtained from^37^; text arrow A indicates the flow direction of Pajaro River; text arrow B and C annotate the tributary of the Monterey Canyon; text arrow D indicates the Soquel Canyon. The green crosses represent the interpreted fault zones around the cable; the gray crosses represented the interpreted paleo-channel unit.

To improve our understanding of the scattered Scholte wave components, we apply a running window FK filter ($100m/s< |v| < 1000 m/s$) along the profile to enhance these weak scattered arrivals as shown in Fig. 4b. We observe that the majority of these scattered arrivals are generated at discontinuity boundaries, particularly at 5.5 km and 9.5 km along the DAS profile. To our knowledge, it is the first time these coherently scattered features have been observed near submarine discontinuities using DAS and an ocean bottom cable; the utilization of such events provides a new approach for characterizing submarine structural features.

### 2D shear wave velocity model

As the lateral discontinuities exist and vary distinctly along the cable, we split the 20 km cable into a series of 1-km-long individual subsections. We obtain 181 Scholte wave shot gathers with the first channel of each subsection as the virtual sources (see Methods). The corresponding middle-point of each shot gather moves from location 1 km to location 19 km. Multimodal phase velocity dispersion curves are generated for each shot gather based on a frequency-domain slant-stacking algorithm, and inverted for 1D shear-wave velocity ($V_s$) structures using the Haskell-Thomson determinant method (see Methods). Fig. 5 shows examples of dispersion measurement and inversion for two Scholte wave shot gathers with virtual sources located at 6 km (Fig. 5a–c) and 17 km (Fig. 5d–f), respectively. We construct a pseudo-2D $V_s$ profile with maximum depth around 350 m based on 181 1D $V_s$ models obtained from all available 1-km-long virtual source gather (Fig. 6). We observe sub-horizontal seabed sediments above 80m depth with shear wave velocity less than 300 m/s, but the lateral velocity discontinuity turns distinct with the depth increasing. In general, we can distinguish four low velocity zones (LVZ) around 5.5 km, 9 km, 15.5 km, and 19 km, and they are consistent with the detected discontinuity boundaries from ambient noise autocorrelation and could be inferred as signatures of potential submarine fault zones. Since the seismic waves can be trapped inside LVZ, it can also explain why we observe stronger Scholte wave energy in these area (Fig. 2a). The high velocity contrasts at partial sections ($2\sim 3\,{\text{km}}; 10 \sim 14 \text{km}; 16\sim 18\,{\text{km}}$) are also consistent with the observation of dispersion measurements where higher modes exist. The inverted 2D velocity structure has been further verified by comparison between the observed waveforms and forward modeling waveforms from an elastic finite-difference simulation; the backscattered surface waves are particularly consistent (see Supplementary Fig. S1).

### Migration of scattered Scholte waves

With the existence of heterogeneities (impedance discontinuities), backscattered surface waves can be generated along the surface, observable as events with moveout in the opposite direction of the indicident surface waves^44,47^. Based on the ambient DAS records, backscattered surface (Scholte) waves have been observed on the retrieved empirical Green’s functions gather (highlighted on Fig. 3a). We utilize these backscattered surface waves to locate the potential scatters or volumetric heterogeneities using two different methods, Kirchoff mapping and natural migration (see Methods). The former utilizes a prior velocity model, while the later uses the natural Green’s function retrieved from ambient interferometry without the knowledge of the velocity model.

Fig. 7a and b show the observed forward-propagating Scholte wave and separated backscattered surface wave around 9.5 km. We build the velocity model (Fig. 7d) based on the converted depth(wavelength)-velocity relationship (indicated by the red dots on Fig. 7c). The velocity model is simplified and represented as a laterally homogeneous media based on the averaged velocities measured from the picked dispersion curves. In order to enhance the imaging coherence, we employ 4 close virtual source gathers as input (indicated by the red stars on Fig. 7d). A continuous energy slope, indicated by the red dash line on Fig. 7e, represents the potential locations of scatterers, and we interpret this slope as a fault dip or structural boundary. The existence of the multiple scattered features, particularly at shallower depths, is caused by spurious arrivals in the retrieved empirical Green’s functions. A synthetic test based on the inverted earth model has been carried out to verify the accuracy of the proposed method (see Supplementary Fig. S2). Compared with the Kirchoff mapping method, natural migration has a lower sensitivity to the quality of the backscattered surface waves because it takes into account multiples, mode conversions and non-linear effects of surface waves in the data^48^. Fig. 8 presents the resulting natural migration image. We observe a distinct zone which scatters Scholte wave energy around location 9.5 km, which is distributed below 200 m depth. Several shallower zones of increased scattering also exist around 3 km, 5 km, 15.5 km, and 19 km. In all of these cases, the zones of Scholte wave scattering can be viewed as geological boundaries with sharp lateral property contrasts.

## Discussion

As we have demonstrated, marine ambient noise recorded by DAS can provide a powerful tool for resolving subsurface property variations at and below the seafloor. Strong noise on the upper side of the microseism band (0.5-10 Hz) recorded by seafloor DAS can be utilized to generate high quality empirical green’s functions; these EGFs can then subsequently be used in a variety of imaging contexts. Scholte wave scattering, detected using FK-filtered EGF autocorrelation profiles, can identify zones with strong lateral property contrasts. Transmitted surface waves retrieved from EGFs can be inverted to generate smooth maps of $V_s$ with sufficient resolution to resolve details in the top 400 m of sediment. By performing wavefield separation, the scattered Scholte waves can then be mapped or migrated to generate a higher resolution image of sharp property contrasts.

Fig. 9 provides an integrated image combining the inversion results from both the transmitted and scattered Scholte wave inversions. As can be seen by from the 750*m*/*s* $V_s$ contour (lowest white line), several low velocity zones (LVZs) exist, including a deep seated anomaly near 9.5 km along the profile. This feature also corresponds to a source of scattered Scholte wave energy as can be seen from the natural migration (background grey scale) and Kirchoff mapping (dashed blue line) results. The zones of scattered energy observed in the filtered autocorrelation profile are shown with the dashed black lines. This combination suggests a zone of reduced velocity with sharp lateral $V_s$ boundaries and vertical extent to at least 400+ m based on the combined results. We interpret the LVZ and associated structure at 9.5 km as an unmapped fault zone, potentially a branch of the AFZ. A zone of decreased velocity and strong lateral scattering, particularly with depth extent, would be consistent with this interpretation. Additionally, there appears to be trapped energy in this zone, visible as persistent higher amplitudes on raw noise gathers, as can be seen in Fig. 2a.

The LVZs identified using Scholte wave inversion located at approximately 15.5 km and 19 km were also confirmed by the natural migration results. They are also likely related to two previously mapped fault zone, one which is part of the AFZ and a second on the eastern edge of the MBFZ, both of which cross the DAS profile, as can be seen in the red lines shown in Fig. 10a. However, these features are also close to shallow paleo-channel features located by^37^, hence there is some ambiguity in this interpretation as will be discussed.

We believe the LVZ near 5 km is more likely to be a deep paleo-channel feature filled with recent sediment; it is directly aligned with outflow of the Pajaro River (the yellow arrow A on Fig. 10a) and the mouth of one Monterey Canyon branch (the yellow arrow B on Fig. 10a). To evaluate our capacity to resolve shallow structural features (top 80 m) we calculated the sensitivity kernels for the Scholte waves at 3 Hz, the center of our bandwidth; the results show that given our noise bandwidth, we have sufficient sensitivity to image shallow (upper 80-m) structural features as can be seen in Supplementary Fig. S3). We should note that the near-surface 250 m/s $V_s$ isocontour, shown in Supplementary Fig. S4, is a good geophysical proxy for recent sediment cover thickness (e.g., the transgressive surface for the seafloor).

As mentioned previously, the shallow (above 80 m) lower velocity (150 m/s) zones $5 \sim 9\,{\text{km}}$ and $14 \sim 16\,{\text{km}}$, compared with the averaged $V_s$ (250*m*/*s*) around the seafloor, could be interpreted as paleo-channel deposits of the Monterey and Soquel canyon systems, respectively. The outline of these two shallow LVZs match well the mapped outlines of paleo-channel unit from high-resolution 2D reflection seismic studies^37^ (the blue dashed lines on Fig. 10a). However, these same reflection studies suggest relatively shallow incised features making them an unlikely source for the deeper $V_s$ structures we have observed using ambient noise. For example, the channels identified by an orthogonal reflection line in^37^ (Fig. 8a in^37^, left feature), close to the 15 km LVZ, have two-way P-wave traveltimes on the order of 0.1 s suggesting maximum depths on the order of 80 m assuming a $V_p$ for seafloor sediment of approximately 1600 m/s^49^. Given the deeper velocity perturbations observed using both transmitted and scattered Scholte waves, there is the possibility that some of these paleo-channel features may be tectonically controlled, with erosion occurring along previously faulted zones. In the same work of^37^, faults in the Purisima formation are noted below some of the channel deposits although their role in channel control is not discussed. Fig. 10b shows our integrated interpretation of the DAS profile in the context of the previously discussed $V_s$ and scattering measurements; the zones of potential fault-related LVZ are shown as green markers while previously mapped faults are shown in red lines; the zone of potential paleo-channel filled with recent sediment is indicated as grey markers around 5 km.

The large spatial scale of the mapped low velocity zones raises the question of what component of fault structure, or channel fill topography, is being interrogated. Refraction tomography and core studies examining seismic velocity variations across the nearby San Gregorio fault^15,50^ show narrower zones of highly reduced velocities, $V_p$ reductions of up to 50%, but over smaller domains of approximately 100 m. In the case of the study by^15^, the fault architecture, initially characterized by^51^, included a narrow gouge core flanked by brecciated materials and a larger zone of highly fractured rock (damage zone). The Aptos and Monterey Bay fault zones have likely not seen the same magnitude of slip as the San Gregorio Fault but there may be a more diffuse set of secondary faults with zones of fracturing but a less developed core. The features resolved using analysis of scattered Scholte waves from our EGFs shows a larger lateral extent in our case, 1-2 km for several of the anomalies. This would be consistent with a sequence of parallel minor faults and their associated damage zones. This hypothesis is partially confirmed by the higher frequency earthquake scattering observations on the same cable discussed in^34^ where a range of local S-to-Scholte wave conversion points are observed in the LVZ zones. The event in question, a strike-slip earthquake (EQ) near Gilroy, CA, was captured by our cable on 11 March 2018 and illuminates the structure directly beneath the DAS cable. As can be seen in Fig. 4, the discrete scattered Scholte waves seen in EGF analysis (panel b) are sufficiently low frequency to obscure the large number of discrete scattering events observed in the regional earthquake record (panel c).

While we have focused entirely on processing of direct and coherently scattered Scholte waves, a variety of other wave modes could be powerful imaging tools for future DAS studies. Strong landward coherent signals of ocean gravity waves can also be observed in lower frequency bands ($< 0.3Hz$) with apparent velocity slower than $\sim$15 m/s from the interferogram (see Supplementary Fig. S5). Analysis of these signals might provide a path to understanding processes in the water column including ocean currents and coastal dynamics. Ambient noise autocorrelation methods have been successfully harnessed to extract reflectors from deep structure in past studies utilizing broadband or short period seismometers^45,46,52,53^. However, this family of techniques has of yet to be succesfully applied to surface DAS data, which tend to be dominated by surface waves. In our context, the extracted autocorrelation signals are most likely Scholte waves rather than reflected S waves considering the strong axial sensitivity of DAS and the horizontal geometry. The high similarity between autocorrelation profile and common offset gather of Scholte wave (see Supplementary Fig. S6) also corroborate this hypothesis. More broadly, the EGFs generated in this study, while of high quality, do not show clear evidence of refracted S wave phases despite extensive processing; this is likely due to a combination of the ambient noise sources, which may not couple efficiently into body waves, as well as sensitivity of DAS to such wave modes. Recent successes in array processing driven by large scale nodal deployments and double-beamforming methods^16^ suggest that future advances may be possible.

DAS provides the powerful combination of high spatial resolution and long spatial profiles. While we process a dataset with 20 km linear extent, advances in photonics are pushing this acquisition distance beyond 100 km (e.g.^54^) which exceeds the mean width of the continental shelf for most margins^55^. As we have shown, the combination of DAS and ambient noise surface wave imaging can be used to generate high resolution depth-resolved profiles of both $V_S$ as well as Scholte wave scattering allowing spatial resolution of features at or below 100 m. Scholte wave scattering in particular may provide a path for resolving small-scale heteogreneities, particularly shallow faults and depositional features near the seafloor, key geohazard mapping targets in many submarine environments.

## Methods

### DAS system installation and cable properties

As previously described in^34^, the DAS interrogator (Silixa iDAS, v.2.3.3.5) was positioned on a passive vibration isolation table in the instrumentation hut where the cable emerges onshore. Connection to the Silixa iDAS was made using an SC-UPC/SC-APC single-mode patch cable. An optical time-domain reflectometer (OTDR) was used to evaluate fiber integrity prior to recording. An OTDR measurement of the optical fiber used for DAS showed 0.19 dB/km of loss over the full fiber length of 52 km with a gauge-length of 10m. A GPS antennae provided accurate timing. DAS data were written continuously via USB 3.0 at 250 MB/s to an external hard disk. In total, 3.2 TB of raw optical phase rate data (proportional to strain-rate) were recorded during the 4 day experiment. The cable used for the MARS umbilical is a single armored submarine cable (Alcatel Submarine Networks, OALC4) including a fiber core with 8 SM fibers surrounded by steel wire strands and an insulating sheath. The outer diameter of the cable package is 31 mm.

### Ambient noise interferometry on DAS records

We utilized ambient noise interferometry to generate the empirical Green’s functions for regularly spaced DAS channels across the array. Before interferometric processing, a sequence of steps were applied to the data to reduce computational expense given the large array size and high temporal sampling. As an initial compression step, we first removed the mean and trend of the dataset in the trace domain followed by band-pass filtering (0.5, 1.0, 40, 80Hz) and temporal decimation (from 1 kHz to 250 Hz). This step was followed by sequential spatial median stacking (5 trace window) and mean stacking (2 trace window) which transformed the dataset from $\sim$10,000 channels with a 2-m spatial sampling interval to $\sim$1000 channels with 20-m spatial sampling interval. This combination of spatial stacking and temporal decimation reduced the dataset size by about a factor of 40. The basic ambient noise data workflow was applied to the continuous DAS dataset (4 days) by processing 1 minute non-overlapping data segments, the native recording unit (strain rate). Preprocessing included mean and trend removal followed by temporal and spectral normalization. Temporal normalization was accomplished using a running absolute mean filter [e.g.^20^]; spectral normalization utilized a frequency-domain whitening approach, which computers the running smoothed amplitude of complex Fourier spectrum as the whiten weights.

We selected every channel from location 0.5 km to 19.5 km as a virtual source, and generated empirical Green’s functions gathers between each virtual source and the whole array (see Supplementary Fig. S7). Next, we performed phase-weighted stacking of all the time segments for each cross-correlation pair to average the effect of temporal noise and spatial irregularity. Finally, we obtain 921 empirical Green’s functions gathers, and each gather includes 1000 channels with a 20-m channel interval. Parts of empirical Green’s functions gathers with the virtual source located near two ends of the cable were not utilized due to strong noise interference.

### Scholte wave dispersion measurement and inversion

For surface wave dispersion analysis, we use 181 empirical Green’s functions with virtual sources located along the array from 0.5 km to 18.5 km. We define the seaward direction as the forward direction in the offset domain (*x*) for each virtual source gather, and select channels with offsets satisfying $0<x<1\,{\text{km}}$ for Scholte wave dispersion analysis (see Supplementary Fig. S7). Finally, we create 181 1-km-long virtual source gathers. The middle-point of each shot gather moves from location 1 km to location 19 km with a regular spatial interval of 100 m. A 1 km array length (*L*) is sufficient to sample a maximum wavelength ($\lambda _{max}$) of up to 300 m ($L > 3*\lambda$)^56,57^, which fulfills our characterization objectives. The high spatial overlap ($90\%$) between virtual source gathers ensures continuity in the inferred 2D velocity structure.

To obtain the Scholte wave dispersion spectra, we apply a frequency-domain slant-stacking algorithm proposed by^58^ to each virtual gather. We first transform the offset-time domain virtual-source gathers into frequency-offset domain representations using a Fourier transform. We then apply a slant-stacking algorithm to construct the dispersion spectra. The energy peaks of the measured dispersion spectra are semi-automatically picked as dispersion curves, which reflects the averaged submarine velocity beneath the 1 km array.

We next invert the dispersion picks for shear wave velocity as a function of depth. To avoid potential mode-misidentification errors in the extracted dispersion curves, we apply a multimodal inversion algorithm which utilizes the Haskell-Thomson determinant method^59,60^ as part of the objective function. It minimizes the determinant of the model-predicted Haskell-Thomson propagator matrix rather than the misfit between observed and forward dispersion curves. Therefore, this inversion algorithm does not require explicit mode labeling, an advantage in DAS datasets where higher overtones are sometimes enhanced.

A Monte Carlo sampling approach is adopted to produce the model pool containing $1 \times 10^5$ models under the predefined search bounds. Note that, a good search bound is crucial for Monte Carlo based inversion given search space exploration constraints. We perform a pre-inversion step to build reasonable search bounds. In this pre-inversion step, we first build loose search bounds (see Supplementary Table 1), and produce the initial model pool for the multimodal inversion; next, we refine search bounds based on the best-fitting models from previous inversion results (see Supplementary Table 2), and produce the final model pool for the multimodal inversion. After this pre-inversion step, we measure the defined misfits for each model and export the final optimal model by misfit-weighted stacking of the best 250 models which posses the lowest misfits. 181 phase velocity dispersion curves were picked and inverted to obtain matching 1D $V_s$ profiles. Finally, we align all available 1D $V_s$ profiles along the cable and build a pseudo-2D $V_s$ image after natural smoothing (a $1\%$-width smoothing factor has been applied on the $V_s$ image along the profile).

### Kirchoff mapping of scattered Scholte waves

Kirchoff migration is a classical seismic migration method to back-propagate seismic wavefield from the region where they are measured into the region to be imaged, by using the Kirchhoff integral representation of wave equation^61^. Backscattered surface waves can be taken as a kind of dispersive reflections observed at surface, and the dispersion character indicates the reflections at different velocity (or frequency) bands are sensitive to scatters at different depths. Based on a prior velocity model, it is possible to map the backscattered surface wave energy to the projection location at the corresponding depth. An appropriate narrow-band filter might contribute to the depth migration imaging result, however, we do not apply it in this context since our effective frequency band is relative narrow ($1\sim 3$Hz).

We first apply FK filter to separate the transmitted surface waves and the backscattered surface waves. Next, we build velocity model based on the measured dispersion curves. In practice, we measure the dispersion curve based on the observed surface wave gather, and convert it into depth(wavelength)-velocity domain using the relationship $depth = \lambda *v/f (0.3<\lambda <0.5)$. Surface waves are dispersive and typically most sensitive to the velocity model to a depth of approximately 1/3 or 1/2 of their wavelength^57,62,63^. In this context, we define $\lambda$ as 0.4. Since the measured dispersion curve is mainly determined by the averaged structure beneath the receiver array^64^, the velocity model is simplified as laterally homogeneous media. For each depth, we apply Kirchoff migration technique to image the horizontal scatters/heterogeneities along the lateral direction based on the simplified earth model and source-receiver configuration. It works like a rotated VSP reflection imaging to locate the reflect/scatter location along the horizontal direction rather than the depth direction. In order to enhance the back-projection energy, we employ 4 virtual source gathers as input shots.

### Natural migration of scattered Scholte waves

Backscattered surface waves can also be imaged for the near-surface heterogeneities based on natural migration using recorded Green’s functions along the surface^48,65^. Natural migration images are evaluated at receivers on the free surface, and they do not directly indicate the depth of the heterogeneities. However, as discussed previously, surface waves provide variable sensitivities with depth for different frequencies, which offers the possibility for frequency-dependent migration images to capture the depth of the heterogeneities. Based on equation 7 on^48^, we simplify the migration equation for backscattered surface wave observed on DAS as

1 $$\begin{aligned} m({\mathbf {x}},\omega _0) \approx -\iiint \omega ^2 \beta (\omega _0,\omega ) \overline{C({\mathbf {x}}|{\mathbf {x}}_s)*C^0({\mathbf {x}}|{\mathbf {x}}_r)}*u({\mathbf {x}}_s, {\mathbf {x}}_r) d{\mathbf {x}}_s d{\mathbf {x}}_r d\omega , \end{aligned}$$

where, $\beta (\omega _0,\omega )$ is the bandpass filter designed to smoothly taper the data and Green’s tensors around the central frequency $\omega _0$; $C({\mathbf {x}}|{\mathbf {x}}_s)$ is the empirical Green’s function observed at source side with virtual source at ${\mathbf {x}}_s$ and receiver at ${\mathbf {x}}$; $C^0({\mathbf {x}}|{\mathbf {x}}_r)$ is the empirical Green’s function observed at receiver side that only contains the transmitted wavefield without backscattering; $u({\mathbf {x}}_s, {\mathbf {x}}_r)$ is the separated scattered wavefield; $m({\mathbf {x}},\omega _0)$ is the scatter image energy at location *x* and frequency $\omega _0$. The wavefield separation is performed using Hilbert transform, which has been frequently used for up/down wavefield separation in reverse time migration^66,67^.

For natural migration, we use total 921 virtual source gathers along the cable with each gather including 1000 channels. In order to save computational effort, we perform the natural migration in the frequency domain and replace the bandpass filter (taper) by applying a median filter ($1\%$ window) on the output natural migration spectrum $m({\mathbf {x}},\omega )$,

2 $$\begin{aligned} m({\mathbf {x}},\omega ) \approx -\iint \omega ^2 \overline{C({\mathbf {x}}|{\mathbf {x}}_s)*C^0({\mathbf {x}}|{\mathbf {x}}_r)}*u({\mathbf {x}}_s, {\mathbf {x}}_r) d{\mathbf {x}}_s d{\mathbf {x}}_r. \end{aligned}$$

Finally, we convert the frequency-dependent scattering image to depth/wavelength based on an averaged dispersion curve from an averaged velocity model beneath the cable.

## Supplementary Information

Supplementary Information 1.

Supplementary Information 2.

## Data Availability

Autocorrelation gathers, empirical Green’s function examples (Fig. 3), picked DAS dispersion curves, Scholte wave inversion results, and scattering reconstructions are available in the following OSF repository: https://osf.io/cn8xb. The earthquake record shown in Fig. 4a is available at Github repository: https://github.com/njlindsey/Photonic-seismology-in-Monterey-Bay-Dark-fiber1DAS-illuminates-offshore-faults-and-coastal-ocean.

## Code availability

Mapped fault zone information was obtained from Quaternary Fault and Fold Database of the United States (last accessed April 2020); The paleo-channel outlines were obtained from Maier et al., 2018; The Gilroy earthquake hypocenter information was obtained from USGS Earthquake Catalog (last accessed April 2020); The transgressive sediment surface dataset was obtained from CaliforniaState Waters Map Series Data Catalog (last accessed June 2020). Computer Programs in Seismology (CPS) package (Herrmann, 2013) was used for surface wave sensitivity kernel calculation; SOFI2D (Bohlen, 2002) was used for 2-D finite difference modelling. Figure 1 is produced by using Generic Mapping Tools (GMT) (last accessed August 2019).
