# Supplementary material for: Utilizing distributed acoustic sensing and ocean bottom fiber optic cables for submarine structural characterization
Source: Sci Rep. 2021 Mar 10;11:5613. doi: 10.1038/s41598-021-84845-y (PMC7946901; doi:10.1038/s41598-021-84845-y)
Supplement: Supplementary file 1 — Supplementary Information 1. [file 41598_2021_84845_MOESM1_ESM.pdf]

Supplementary materials for
Utilizing Distributed Acoustic Sensing and Ocean Bottom Fiber Optic Cables for Fault Zone Characterization

Cheng et al.

Elastic finite-difference modeling: waveform comparison

In order to verify the accuracy of the inverted velocity model, we generate a synthetic shot gather using a finite-difference solver, SOFI2D [1], to allow direct comparison of various wave modes. We utilize the 2D V_s model recovered from Scholte wave inversion. A horizontal force with source signature defined by the EGF autocorrelation function at location 8.2 km is used as the source input function. We use a grid spacing of $2m$ in both X and Z to avoid numerical dispersion. A time step of $50\mu s$ is used to guarantee model stability. Fig.S1 shows a direct comparison of the modeled shot gather (red) and the ambient noise EGF measured using DAS (blue). As can be seen, the gathers compare relatively well, particularly the observed backscattered surface waves (right panel). This result bolsters our confidence in the recovered velocity model. However, some local differences, particularly around location 9.8 km, are apparent. This is likely due to the use of a smooth V_s model which is known to be incorrect based on the natural migration results. A second factor is that we do not explicitly consider the water-solid interface effects but simplify the problem using a free surface boundary condition.

Scattered Scholte wave mapping: numerical test

We peformed a series of numerical tests to evaluate the feasibility of applying Kirchoff mapping to backscattered surface waves. We extract two averaged velocity models from the inverted earth model at locations 9.0 km and 10km and constructed a simple fault model with known dip (Fig.S2a). Next, we generated a synthetic shot gather (Fig.S2b) using a 3 Hz Ricker wavelet as a source and the elastic finite difference simulator discussed previously (SOFI2D). The source and receiver array configuration is shown by the star and triangle in Fig.S2e. Fig.S2c shows the backscattered surface (Rayleigh) waves from the fault, separated in the FK domain. In order to build a depth-velocity relationship, we measure the dispersion curve (blue dots on Fig.S2d) based on the observed surface wave (only the blue shadow zone), and convert the picked dispersion curve into wavelength(depth)-velocity profile (black circles on Fig.S2d) using $depth = 0.4 * v/f$. Based on the extracted depth-velocity relationship, we build a series of homogeneous models for all available depths. Fig.S2e shows the three velocity models at depth $50m$, $90m$, $122m$ using the corresponding velocity measured on the wavelength(depth)-velocity profile (indicated by the red circles on Fig.S2d). We apply Kirchoff mapping to the separated backscattered surface waves to image the horizontal heterogeneities for each depth based on the corresponding laterally homogeneous velocity model. Finally, we combine the back-projection image along the depth direction to track the locations of the fault. Fig.S2f displays the stacked migration image and the distinct energy peaks match the true fault location well, which indicates the feasibility of this technique for mapping horizontal heterogeneities characterization. The biases below depth $120m$ are caused by the weak sensitivity of the observed backscattered surface waves. We should note that we are only considering the 2D X-Z plane in this case, where the fault is normal to the profile with a single fixed dip. For profiles with multiple orientations and better coverage, explicit consideration of fault azimuth could also be considered.

Shallow sedimentary structure characterization

In order to check the sensitivity of the observed Scholte waves, we computer the sensitivity kernel (Fig.S3) of the fundamental mode surface wave based on the inverted earth model using the Computer Programs in Seismology (CPS) software package [2]. Fig.S3 shows that the observed Scholte wave are highly sensitive to the shallow submarine sediment layers.

We integrate our inverted V_s model with the documented sediment maps from California State Waters Map Series [3]. The sediment maps (transgressive surface for the seafloor) is interpreted by high-resolution seismic-reflection data supplemented with outcrop and geologic structure. Fig.S4 show a reasonable match between the mapped sediment transition depth and our inverted V_s model for the upper layers of the model. The upper panel on Fig.S4 shows the cross-section profile (red line) along the cable line match well with the shallow V_s distribution, particularly at $250m/s$ V_s contour (white line). It indicates that our observation is able to provide a supplementary on the shallow sediment structural features characterization.

Coherent signal retrieval for ocean surface gravity wave

We utilize classical ambient noise interferometry techniques to generate empirical Green's functions by cross-correlating pre-processed DAS records at different channel. Compared with Scholte waves, ocean surface gravity waves usually possess lower frequency between 0.1 Hz and 0.3 Hz. In order to retrieve the coherent signal of ocean surface gravity waves, we need focus on the primary microseism. The data processing workflow is almost the same as that for Scholte wave retrieval, except that we apply a different bandpass filter parameter (0.05, 0.1, 0.3, 0.5 Hz) in the initial preprocessing step. Fig.S5a presents an example of the retrieved coherent signal for ocean surface gravity wave with virtual source at 1.32 km location. We can observe clear land-ward coherent signals with apparent velocity around $15m/s$. Fig.S5b shows the corresponding frequency-wavenumber (FK) spectrum. The dispersion curve associated with the strongest FK energy obeys the dispersion relationship of the linear gravity wave theory [4, 5]

$$\omega^2 = gktanh(kH) \tag{1}$$

, where ω is angular frequency, g is gravitational acceleration, $k$is wavenumber, and H is water depth.

Reference

[1] Thomas Bohlen. Parallel 3-d viscoelastic finite difference seismic modelling. *Computers & Geosciences*, 28 (8):887–899, 2002.

[2] Robert B Herrmann. Computer programs in seismology: An evolving tool for instruction and research. *Seismological Research Letters*, 84(6):1081–1088, 2013.

[3] S.Y. Johnson, S.R. Hartwell, R.W. Sliter, J.T. Watt, and K.L. Maier. Sediment thickness—pigeon point to monterey, california, in golden, n.e., compiler, 2013. California State Waters Map Series Data Catalog: U.S. Geological Survey Data Series 781, 2016. URL https://pubs.usgs.gov/ds/781/.

[4] H Lamb. Hydrodynamics, 6th edn new york. *NY: Dover Publications.[Google Scholar]*, 1945.

[5] A. Sladen, D. Rivet, J. P Ampuero, L. De Barros, Y. Hello, G. Calbris, and P. Lamare. Distributed sensing of earthquakes and ocean-solid Earth interactions on seafloor telecom cables. *Nature Communications*, 10 (1):5777, dec 2019. doi: 10.1038/s41467-019-13793-z.

Layer number	$\mathbf{V_s}(km/s)$	ν	$\boldsymbol{\rho}(g/cm^3)$	$\mathbf{h}(m)$
1	(0.1, 3.5)	(0.2, 0.5)	(2.0, 2.0)	(20, 70)
2	(0.1, 3.5)	(0.2, 0.5)	(2.0, 2.0)	(20, 70)
3	(0.1, 3.5)	(0.2, 0.5)	(2.0, 2.0)	(20, 70)
4	(0.1, 3.5)	(0.2, 0.5)	(2.0, 2.0)	(20, 70)
5	(0.1, 3.5)	(0.2, 0.5)	(2.0, 2.0)	(20, 70)
Half-space	(0.1, 3.5)	(0.2, 0.5)	(2.0, 2.0)	(20, 70)

Table 1: Parameters of initial search bounds for Scholte wave inversion. V_s denotes the shear wave velocity; V_p denotes the compressional wave velocity; ν and h indicate the Poisson's ratio and thickness. The values inside bracket indicates the lower and upper bounds of specific parameter at each layer.

Layer number	$\mathbf{V_s}(km/s)$	ν	$\boldsymbol{\rho}(g/cm^3)$	$\mathbf{h}(m)$
1	$V_s^{best} + (-0.2,\ 0.3)$	ν^{best}	2.0	$h^{best} * (0.5,\ 1.5)$
2	$V_s^{best} + (-0.2,\ 0.3)$	ν^{best}	2.0	$h^{best} * (0.5,\ 1.5)$
3	$V_s^{best} + (-0.2,\ 0.3)$	ν^{best}	2.0	$h^{best} * (0.5,\ 1.5)$
4	$V_s^{best} + (-0.2,\ 0.3)$	ν^{best}	2.0	$h^{best} * (0.5,\ 1.5)$
5	$V_s^{best} + (-0.2,\ 0.3)$	ν^{best}	2.0	$h^{best} * (0.5,\ 1.5)$
Half-space	$V_s^{best} + (-0.2,\ 0.3)$	ν^{best}	2.0	$h^{best} * (0.5,\ 1.5)$

Table 2: Parameters of refined search bounds. V_s^{best} denotes the best fitted shear wave velocity; ν^{best} and h^{best} indicate the best fitted Poisson's ratio and thickness. The values inside bracket indicate the adjustment applied on the best fitted models, which lead to the refined lower and upper bounds.

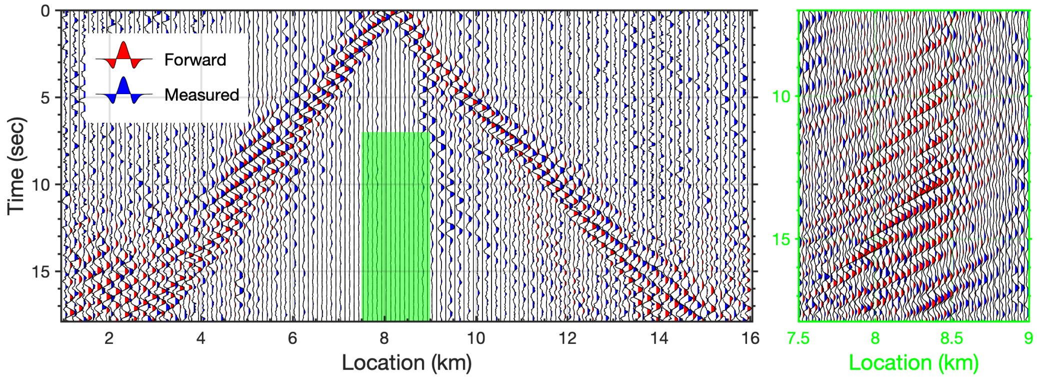

Figure S1: Comparison between the forward modeled waveforms, based on the inverted V_s model, and the observed Scholte waves recovered from ambient noise interferometry. The left panel shows the zoomed window after time power gained ($t^{0.7}$). The red color filled traces represent the forwarded waveforms; the blue color filled traces represent the measured waveforms.

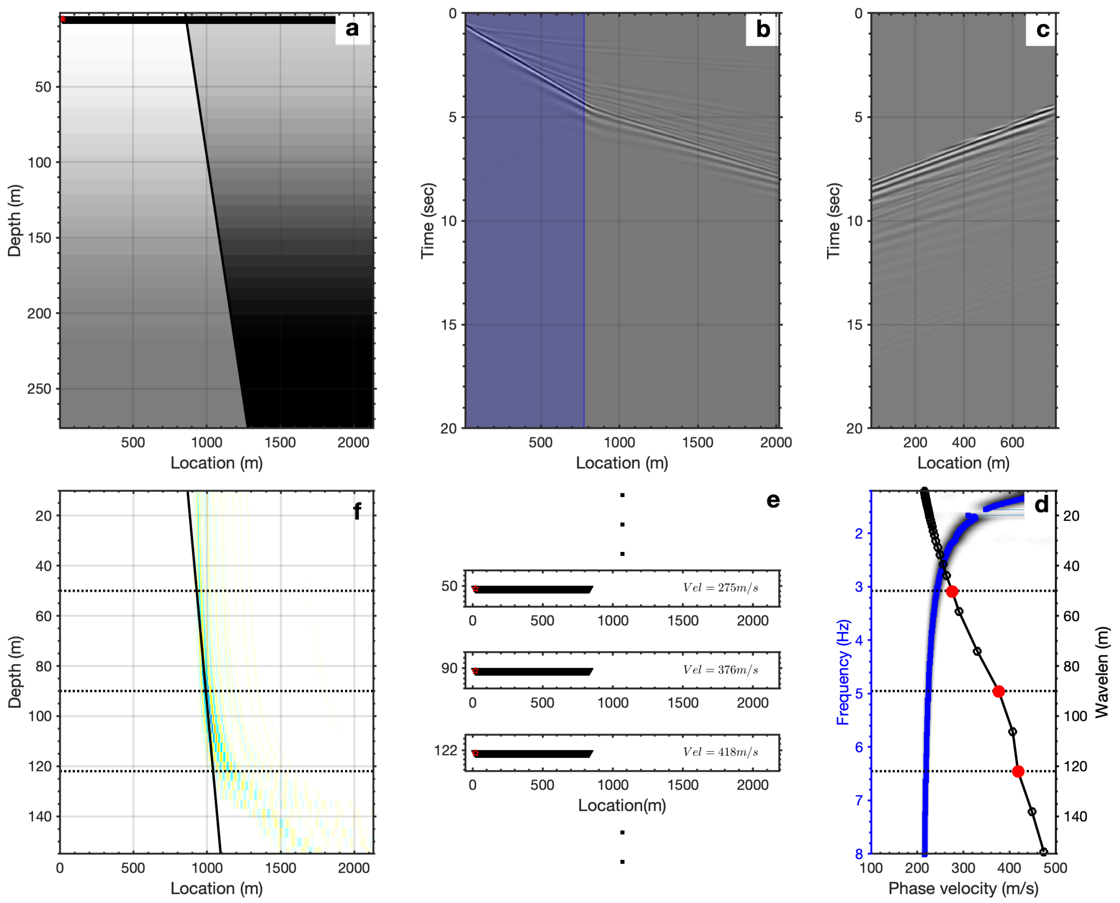

Figure S2: Test example of backscattered surface wave mapping. a). the fault velocity model; b). the synthetic shot gather; c). the separated backscattered surface waves; d). the measured dispersion spectra and the converted wavelength(depth)-velocity profile; e). input velocity models for Kirchoff migration at different depths; f). the stacked migration image compared to the true fault location and dip. The source and receiver array configuration is indicated by the red star and black triangles. The blue shadow zone on panel b indicates the section where backscattered surface waves can be observed and where the surface wave gather is used for dispersion measurement.

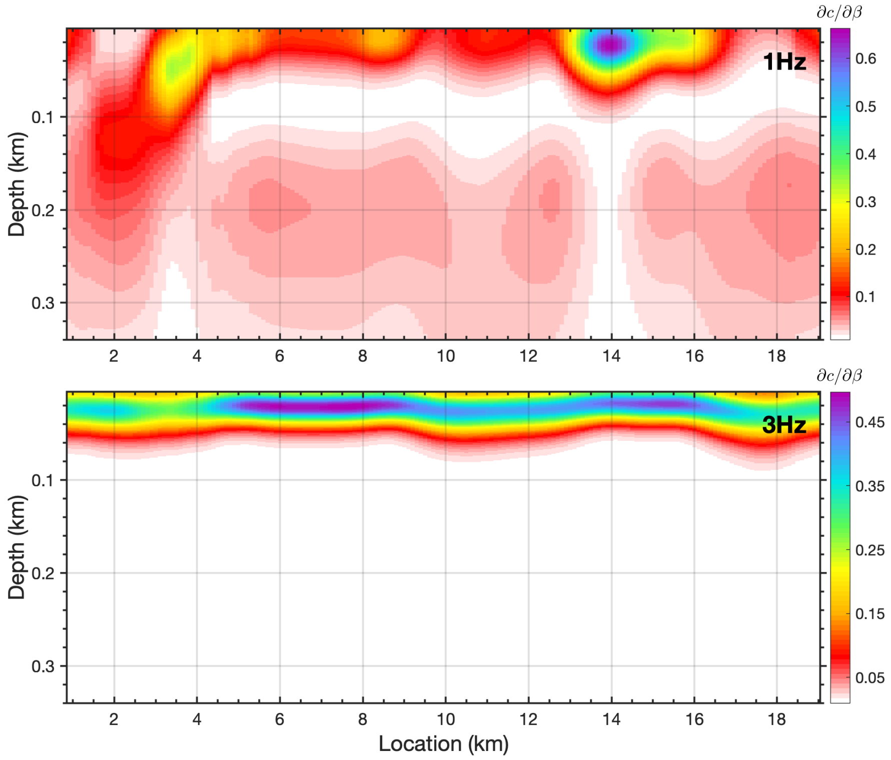

Figure S3: Sensitivity kernels of the fundamental mode surface wave based on the inverted earth model for 1 (top) and 3 (bottom) Hz.

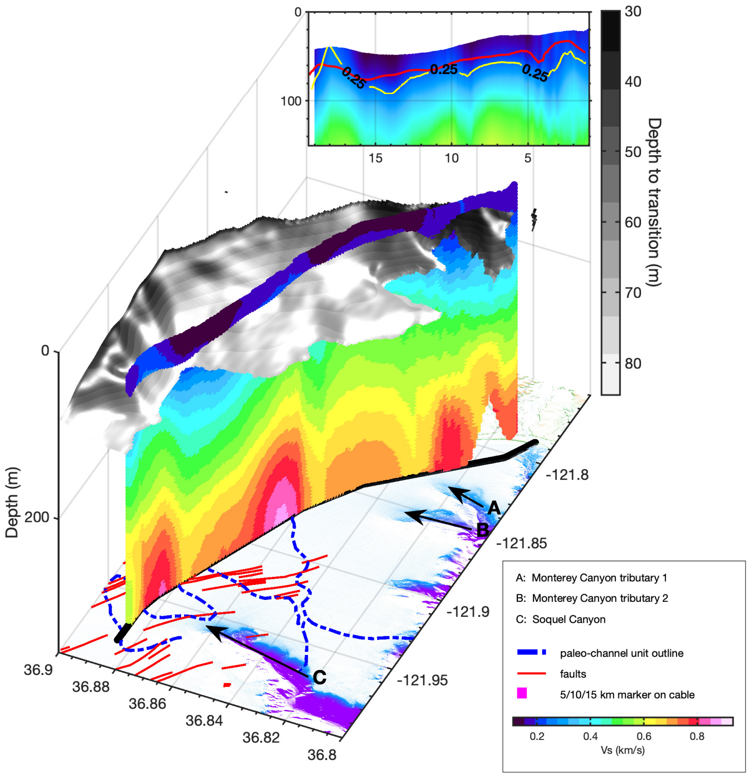

Figure S4: Shallow sediment structure comparison. The grey image shows the mapped transgressive surface from California State Waters Map Series. The vertical color image shows the inverted V_s profile from ambient noise Scholte wave analysis (Fig.**??**). The upper panel shows a cross-sectional profile (the red curve) between transgressive surface and V_s slice, which matches the V_s contour at $250m/s$ reasonably well (the yellow curve). The three pink squares on the V_s profile indicate marks for location $5km$, $10km$, $15km$; The dark red lines represent the mapped faults; the blue dashed lines indicate the outline of paleo-channel units. Text arrow A and B annotate the tributary of the Monterey Canyon; text arrow C indicates the Soquel Canyon.

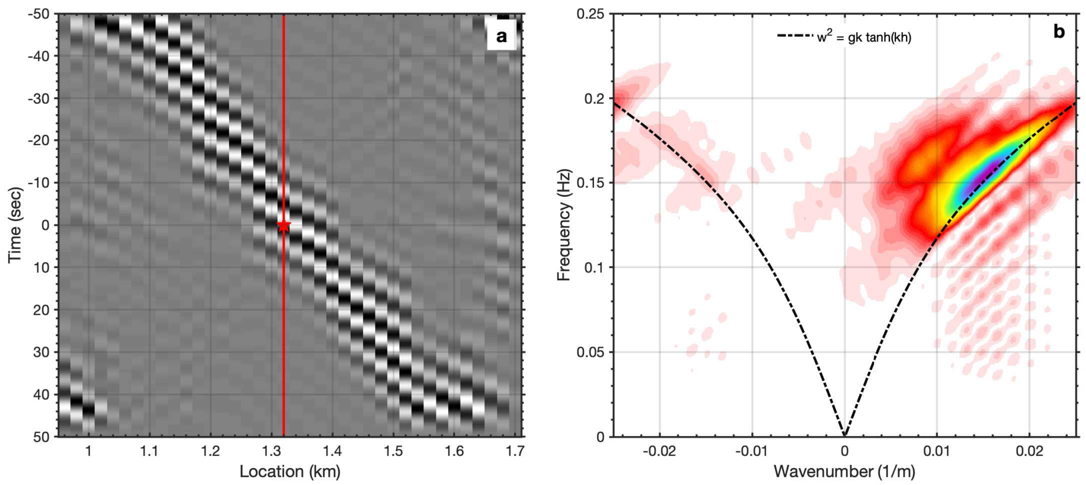

Figure S5: Retrieved coherent signal for ocean surface gravity wave at location around 1.3 km.

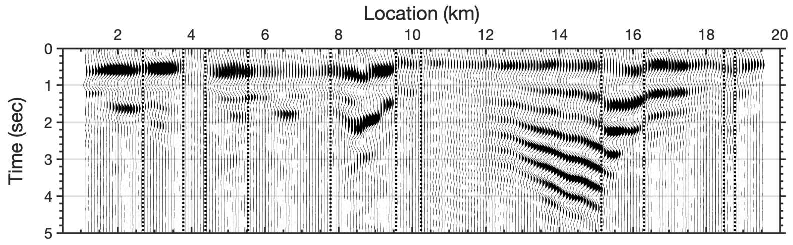

Figure S6: Common offset gather from the retrieved Scholte wave along the cable (offset 300-meter). The black dashed lines indicate the detected horizontal discontinuity boundaries from autocorrelation image.

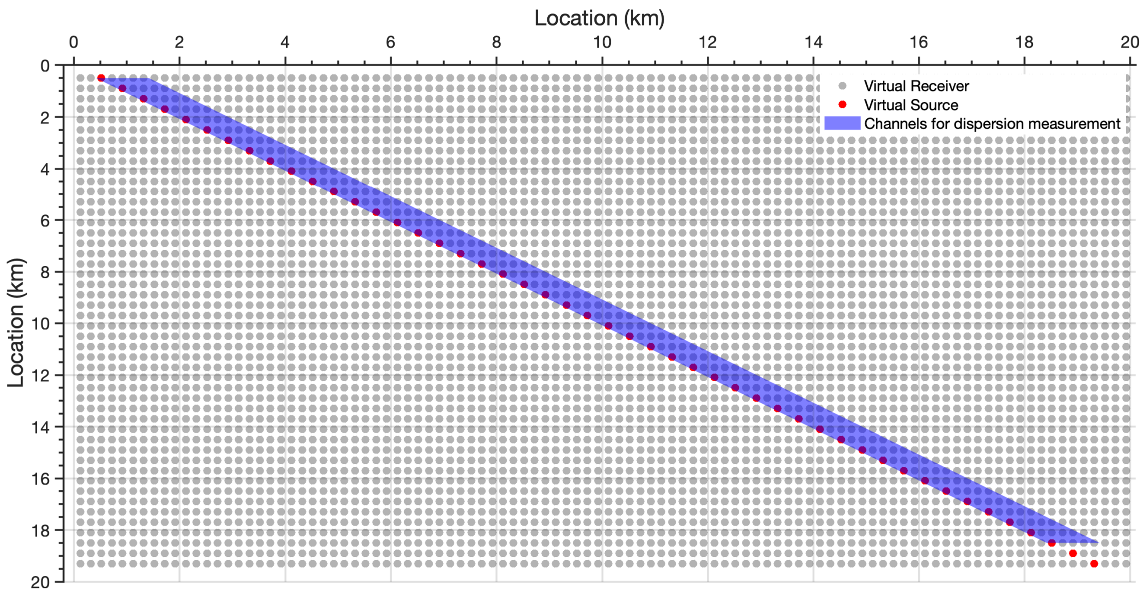

Figure S7: Virtual source and receiver configuration for ambient noise interferometry.
